# Hypnotism and Suggestion

**Published:** 1893-12

**Authors:** 


ARTICLE VIII.

### HYPNOTISM AND SUGGESTION.

The interesting experiments carried out by Krafft-Ebing, at a recent meeting of the Vienna Society for Psychiatry and Hypnotism, have aroused great interest in scientific circles. The experiments were made with the object of showing that it is possible, by hypnotic suggestion, to transfer persons into a former period of their lives, their mental condition, at the same time, undergoing a corresponding change, and that while in this state nothing is lost to their memories which cannot, by suitable influence, be recalled. The astonishing results obtained with Miss P. by hypnotic suggestion are the more seriously considered, because brought to notice by so learned an observer as Krafft-Ebing. The subject was a woman of 33 years of age. Krafft-Ebing hypnotized her, and transferred her successively back to the ages of 7, 15 and 19, restoring her, after each experiment, to her normal condition. In each case she behaved, spoke, and wrote in a way corresponding to the age which she imaged herself to be. The experiments were received, by the majority of those present at the meeting, with much skepticism. In decided contrast with the opinions of certain psychologists and neurologists, who, while they doubt the genuineness of the three states shown in Miss P. by the demonstrator, still admit their possibility, stand the views of Professor Benedikt, who addressed Krafft-Ebing's hearers in a way quite characteristic of his energetic manner: "Well, the whole thing is a swindle. The possibility that such things may be presented before a scientific assembly, and that they are not at once recognized as a clumsy deception, is accounted for by the fact that the physicians and psychologists present do not possess a true knowledge of humanity, and are unable to make a correct analysis of such proceedings. One of the most interesting subjects of psychology is the fact that intellectual, physically and morally sound women are not those who are fascinating to men, but, on the contrary, the hysterical women; this results from the fact that the hysterical women presents the feminine type, even to the verge of caricature, and, according to the law of contrast, calls forth the admiration of the masculine sex. These hysterical women also have the feminine peculiarity of profiting by the intellectual, moral, or æsthetic weaknesses of the male sex. It is, therefore, a great satisfaction to them to get the better of any enthusiast, 'poseur,' or learned man. When we also consider that idle, lazy members of the higher classes of society require a special science for themselves, which they can pursue without labor or special knowledge, it becomes evident why medical doctrines and occurrences which tickle the fancy and self-sufficiency of these idlers so easily become the fashion. The teachings of Kneipp are naturally more accessible than those of Skoda, and these gentlemen can amuse themselves with all the fantastic nonsense of modern hypnotism and modern suggestion, and look down upon their more-learned fraternity, who do not accept such vague and uncertain theories. I belong to those who have long been occupied with the study of hypnotism, and have used it as a curative agent. If I did not immediately decry the exaggerations originating at Nancy, it was because frequently, in medicine, the truth has only left its impress to posterity clothed in a heavy garb of errors. The phantoms and the humbug pursued for years I have combated with success in Paris, in 1889; in Bournmouth, at the meeting of English physicians, in 1890; and in Brussels, the same year. The misapprehensions into which some noble minds, such as those of Charles Richter and Luys, have fallen stand to-day under the judgment-sword of the scientific world, which not only combats hypnotism, but warns and protects itself against its exaggerations and misuses.

It may be here stated that this form of suggestion is not particularly new. Seven years ago, similar experiments were made in Finland by Prof. Karl Hansen, of Copenhagen, who, later on, also made further experiments of the same kind. Hansen transferred the subject to a period of old age, and it was most interesting to note how the posture and even the facial expression corresponded with the period suggested. In the Paris and Berlin scientific circles the Vienna experiments call forth much ominous headshaking. The opinion seems to be pretty general that Krafft-Ebing has been made the victim of a very cleverly perpetrated deception,—by no means the first.—*Ed. Univ. Med. Journal.*

